# Menstrual cycle or hormonal contraceptive related symptoms in elite female athletes from retrospective self-questionnaires and daily monitoring: impact on well-being and objective performance metrics

**DOI:** 10.3389/fspor.2025.1693190

**Published:** 2025-11-25

**Authors:** Nolwenn Badier, Marine Dupuit, Tom Chassard, Kilian Barlier, Alice Lafitte, Guillaume Saulière, Lidia Delrieu, Jean-François Toussaint, Juliana Antero

**Affiliations:** 1 UMR 7329—Institute for Research in Medicine and Epidemiology of Sport (IRMES), Université Paris Cité, National Institute of Sport, Expertise and Performance (INSEP), Paris, France; 2 Center for Investigation in Sport Medicine, CIMS Hôtel-Dieu, Assistance Publique—Hôpitaux de Paris, Paris, France

**Keywords:** menstrual cycle, athletes, survey and questionnaire, monitoring, athletic performance, menstrual symptoms

## Abstract

**Introduction:**

Negative menstrual cycle–related symptoms have been shown to perceptually impact well-being, as well as training and competition performance, yet they are rarely daily monitored over extended periods. The aim is to compare menstrual cycle (MC) and hormonal contraception (HC) related symptoms reported through retrospective and daily questionnaires in elite female athletes and to assess their association with well-being and performance metrics.

**Methods:**

Data were collected from 108 elite female athletes across seven sports over 554 full cycles. Participants completed a retrospective questionnaire on regular symptoms and provided daily prospective entries for 6 months (*n* = 16,491) about their symptoms, sleep, fitness and mood. Symptom reporting methods were compared, differences in symptom frequency across cycle phases were analyzed, and associations between symptom count and well-being were explored. Performance metrics were collected for sports providing objective data and analyzed comparing training with vs. without reported symptoms.

**Results:**

Athletes reported more symptoms retrospectively than in daily questionnaires. Mood swings, tiredness, and pelvic pain were the most common retrospective symptoms, while bloating, tiredness, and pelvic pain were most frequent in daily entries. MC athletes reported more symptoms than HC users in both methods. According to the daily follow-up, symptoms were more frequent during menstruation and the prebleeding phase for MC athletes and the break phase for HC users. A significant negative correlation was observed between symptoms and well-being indicators. In football players, high-speed running distance significantly declined on symptomatic days.

**Conclusion:**

Retrospective questionnaires shows a greater symptom prevalence than daily monitoring. Symptoms, more frequent during bleeding phases, negatively impacted athletes' well-being and also high-speed running performance for football players. These findings highlight the importance of individualized monitoring and tailored interventions to optimize athlete health, well-being, and performance.

## 1 Introduction

The menstrual cycle (MC), characterized by cyclical fluctuations in sex hormones, may influence well-being and sporting performance through a wide range of physiological and psychological responses (1). Although MC characteristics and symptomatology are highly individual, a recent review highlighted that MC-related symptoms are commonly reported in female athletes particularly during menstruation and the premenstrual phase (2): 24%–100% of athletes report experiencing physical and/or psychological symptoms. Negative MC-related symptoms have been shown to perceptually impact well-being as well as training and competition performance (3–8), particularly during and just before menstruation (2), when their prevalence is highest.

An important methodological question that remains is how to best collect data on menstrual symptoms. Most studies in athletes have relied on retrospective questionnaires or semi-structured interviews (2). Such methods do not allow quantification of symptom frequency or cyclicity and are prone to memory bias (9). This bias refers to the discrepancy between what individuals actually experience in real time and what they later remember and report. In contrast, daily questionnaires, which have been used in some studies (10–12), provide data that are closer to real time, reduce recall bias, and offer a more sensitive assessment of symptom fluctuations, including the possibility of estimating their frequency and cyclicity. Despite their potential, no study has directly compared symptom reports collected retrospectively vs. longitudinally, leaving it unclear whether the method of data collection influences the conclusions drawn.

Finally, hormonal contraception (HC) methods are commonly used in athletic population particularly oral contraceptives (13, 14). Indeed, in addition to birth control, HCs are sometimes prescribed to alleviate MC-related symptoms (15). However, HC use can also be associated with some negative side effects/symptoms such as headaches, mood changes or tiredness (14, 16–18). Despite the more stable hormonal profile resulting from the downregulation of endogenous hormones (19), athletes using HCs still report that their performance or well-being may be affected when these symptoms occur (14). For users of combined oral contraceptives, the standard regimen alternates between three weeks of active hormone intake (“active phase”) and one week without hormones or with placebo pills (“pill-break phase”). This schedule can therefore be considered an intermittent HC regimen. In contrast, some HC methods (e.g., hormonal IUDs, implants, or extended/continuous oral contraceptive use) provide uninterrupted hormonal exposure and can be considered continuous HC regimens. Few studies have investigated how athletes' perceptions of symptoms and performance may differ between the active and pill-break phases of intermittent regimens. Even fewer have compared intermittent and continuous HC use more broadly.

Another important gap concerns the impact of these symptoms on objective performance, which is particularly relevant for elite female athletes, whose body is their primary tool for success. Optimal performance is essential for securing titles and medals, which in turn bring visibility, financial rewards, sponsorship opportunities, and career advancement.

A better understanding of menstrual symptoms across the MC, whether in regular or irregular cycles, as well as across intermittent and continuous HC use, in terms of type, frequency, and recurrence, is needed to improve the support provided to athletes. In addition, clarifying how different methods of symptom collection (e.g., retrospective vs. daily monitoring) influence the results is essential.

Therefore, the first aim of this study was to compare symptoms related to MC or HC reported in a retrospective questionnaire with a daily monitoring. Our second aim was to describe symptom occurrences during regular or irregular MC, and during intermittent or continuous HC phases. Then, we investigate if menstrual symptoms have an impact on athletes' well-being and on objective metrics of performance. We hypothesized that symptoms would be underreported in retrospective questionnaires compared to daily monitoring, as daily tracking is more sensitive to short-term variations. We further hypothesized that symptom occurrence would differ in symptom occurrence between cycle types, with more frequent and severe symptoms in irregular compared to regular cycles, and during menstruation or the pill withdrawal phase compared to active phases or continuous HC use. Finally, we hypothesized that a greater number of symptoms would be associated with lower well-being and decreased performance metrics.

## 2 Methods

### 2.1 Design

This study relied on a retrospective survey and a prospective longitudinal follow-up of an elite female athletes' cohort. Prior to participation, all athletes were informed about the purpose of the study and collected data, then they signed a consent letter. All investigations conformed to the code of ethics of the World Medical Association (Declaration of Helsinki) and were approved by the Institutional Ethics Committee (IRB00012476−2022-03-11-206). Data collection complied with the General Data Protection Regulation (2016/679) applied in the European Union and received a certificate of compliance from the Commission Nationale Informatique et Libertés (CNIL-2221532).

### 2.2 Participants

Data from 109 French elite female athletes—tiers 4 and 5 of the classification framework for research in sport (20)—were collected from February 13, 2021 to July 8, 2024. The athletes, preparing for the Olympic and Paralympic Games, practiced cycling, rowing, skiing, football, swimming, triathlon and wrestling. Both naturally menstruating athletes (MC athletes), including copper-based intrauterine devices (IUDs) users (21), and hormonal contraception users (HC athletes), whether using intermittent or continuous contraception, were included. Athletes were informed about the study and voluntarily agreed to participate for a minimum of 6 consecutive months of daily follow-up during the study's period.

### 2.3 Retrospective and daily questionnaires

A meeting was held with the athletes to provide all information about the study protocol. Volunteering athletes were asked to complete an initial questionnaire, previously described (22), to collect demographic information, details about their training and competition levels, menstrual history, and retrospective self-perceived cycle-related effects on performance. Athletes had to select frequently experienced symptoms (i.e., at least 50% of their cycles) related to their MC or HC use, from a list of 12 physical and physiological symptoms commonly described in the literature as being frequently experienced during the MC (2, 4, 23): bloating, breast pain, cravings, digestive troubles, pelvic pain, headaches, heavy bleeding, hypotension, mood swings, soreness, tiredness, and water retention.

Athletes were then monitored using a daily questionnaire administered through a smartphone application created for this research (22). Participants reported the start and end of menstruation for MC athletes, or the start and end of the break phase (inactive hormonal contraception days) for intermittent HC users. Each day, athletes were asked to complete the questionnaire, indicating whether they had experienced any of the 12 menstrual symptoms listed in the retrospective questionnaire on that day or the previous day.

In both questionnaires, athletes could indicate additional symptoms not listed among the 12 items.

### 2.4 Cycles moments definition

A cycle starts on the first day of menstruation or the HC break and ends the day before the next menstruation or HC break. For MC athletes, the cycles were classified into 3 phases: (i) menstruation phase: days of bleeding; (ii) in-between phase: between menstruation and prebleeding phase; (iii) prebleeding phase: backward 7 days from the onset of the next menstruation (24). Each cycle was then classified according to its duration: a regular cycle was defined as having a length between 21 and 35 days (25), and a cycle-to-cycle length variation fewer than 7 days (26, 27). Other cycles were defined as irregular. For athletes using intermittent HC, two phases were determined: (i) active hormonal phase and (ii) break phase (22). For athletes using continuous HC, cycle lengths were arbitrarily fixed at 28 days and no phase division was applied.

### 2.5 Well-being and performance parameters

In addition to the menstrual symptoms, the daily monitoring was used to track well-being parameters: sleep quality, fitness, and mood using a Likert scale ranging from 1 to 10 (22).

For female football players and cyclists, objective performance parameters were obtained from sensors worn during trainings. In football, metrics were collected via Global Positioning System (GPS) including the total distance covered (in meters.min^−1^) and distances covered at various speed intervals: 0–6, 6–13, 13–19, 19–23, and over 23 kilometers per hour. Each distance covered at these speeds was divided by the total training distance, to obtain the proportion of total training distance covered at each speed ranges. In cycling, normalized power output has been used and calculated as follows (28):

1. Excluding the first 30 s of training, a 30 s moving average from the power recorded on the bike sensors is calculated:
  $$\bar{P}[i]=\frac{1}{30}\sum_{i}^{i+30}P[i]$$

with $\bar{P}$ the 30 s moving average, *P* the power in watts from the bike's sensors, and *i* the time at which the moving average is calculated (*i* = 30 for the first moving average).

1. The normalized power output is then calculated:
  $$\mathrm{NP}={\left(\frac{1}{T-30}\sum_{i}^{T}{\left(\bar{P}[i]\right)}^{4}\right)}^{\frac{1}{4}}$$

with T the total duration of the workout.

Training sessions with outlier's metrics, defined as measures less than or greater than the mean plus two standard deviations, were removed for both football and cycling.

The performance of the other sports included in the cohort could not be objectively analyzed due to the unavailability of *in situ* sensors.

### 2.6 Data analyses

Data from the retrospective questionnaire were binary showing whether an athlete regularly experienced each symptom or not. The daily questionnaire data were collected for 6 months, allowing athletes to report each day whether they experienced any specific symptom. To compare these two datasets, a “frequent symptom” in the daily questionnaire was defined in the same way of the retrospective questionnaire, as one that occurred in more than 50% of the athlete's tracked cycles—that is, if the symptom was reported at least once during more than half of the recorded cycles. A new dataset was then created, where each symptom was represented as a binary variable: 1 if it met the criteria for a regular symptom, and 0 if it did not. This allowed the two datasets to be compared.

For both questionnaires, the number and proportion of athletes who frequently experienced each symptom was described. A Wilcoxon test was performed to assess the differences between MC and HC groups. McNemar's test was then applied to determine the differences between the number of athletes reporting a symptom in the retrospective questionnaire and those reporting the same symptom in the daily questionnaire (29).

For each symptom, the proportion of symptomatic days for each athlete in each cycle was then calculated by dividing the number of days with symptoms by the total number of days in that cycle. This proportion was then multiplied by 28, to determine the number of days on which the athlete reported a symptom during a typical 28-day cycle, and described for regular and irregular MC, and intermittent and continuous HC. To compare the occurrence of symptoms across cycle types, linear mixed-effects models were fitted with cycle type as a fixed factor and athlete as a random effect, accounting for repeated measures among athletes who contributed data to multiple cycle categories. For each symptom, a global *p*-value for the effect of cycle type was computed, followed by pairwise *post-hoc* comparisons adjusted using the Holm method. Similarly, the proportion of symptomatic days for each phase of each cycle was calculated. Symptom's frequencies differences between MC phases (menstruation vs. in-between vs. prebleeding)—depending on regular and irregular cycles—were assessed using a Friedman test, followed by a Wilcoxon paired *post-hoc* test. Differences in symptom frequency between intermittent HC phases (active hormonal vs. break) were evaluated using a Wilcoxon paired test. All tests were adjusted with Holm-Bonferroni method.

Spearman's correlations were used to evaluate the link between the number of symptoms reported and the evaluation of well-being parameters. The association between symptoms and well-being parameters was analyzed using a Wilcoxon test to assess indicators when an athlete reported at least one symptom vs. when she reported none.

A Wilcoxon test was used to assess performance metrics when an athlete reported at least one symptom vs. when she reported none.

Missing data i.e., days when athletes did not respond to the questionnaire, were not analyzed. For all analyses, the threshold of significance was defined as *α* = 0.05, and R software was used (Version 4.3.3, R Foundation for Statistical Computing, Vienna, Austria).

## 3 Results

### 3.1 Participants

One athlete interrupted the use of HC and was excluded from the analysis. A total of 108 athletes participating in 7 sports: cycling (*n* = 31, 28.7%), rowing (*n* = 5, 4.6%), skiing (*n* = 9, 8.3%), football (*n* = 33, 30.6%), swimming (*n* = 9, 8.3%), triathlon (*n* = 7, 6.5%), and wrestling (*n* = 14, 13.0%), were retained.

MC athletes represented 58.3% (*n* = 63) including 6 copper IUDs users. The contraceptive methods used by HC athletes (*n* = 45, 41.7%) were predominantly combined HC (*n* = 36, 80.0%). Specifically, 31 athletes used monophasic pills, 1 used biphasic pill, and 3 used triphasic pills, while another one used a vaginal ring. Three athletes used only-progestative pills and 6 used continuous HC.

Monitoring dataset encompasses a total of 554 full cycles, with an average 5.1 ± 1.6 cycles per athletes during the follow-up period, totaling 16,491 daily self-reported entries and a daily response rate of 88.9%.

### 3.2 Retrospective questionnaire

Among all athletes, the average number of symptoms retrospectively reported as frequent (i.e., occurring at least once per cycle in more than 50% of tracked cycles) was 3.5 ± 2.6. MC athletes reported significantly more symptoms (4.0 ± 2.7, *p* = 0.037) than athletes using HC (2.9 ± 2.3).

**Figure 1:**
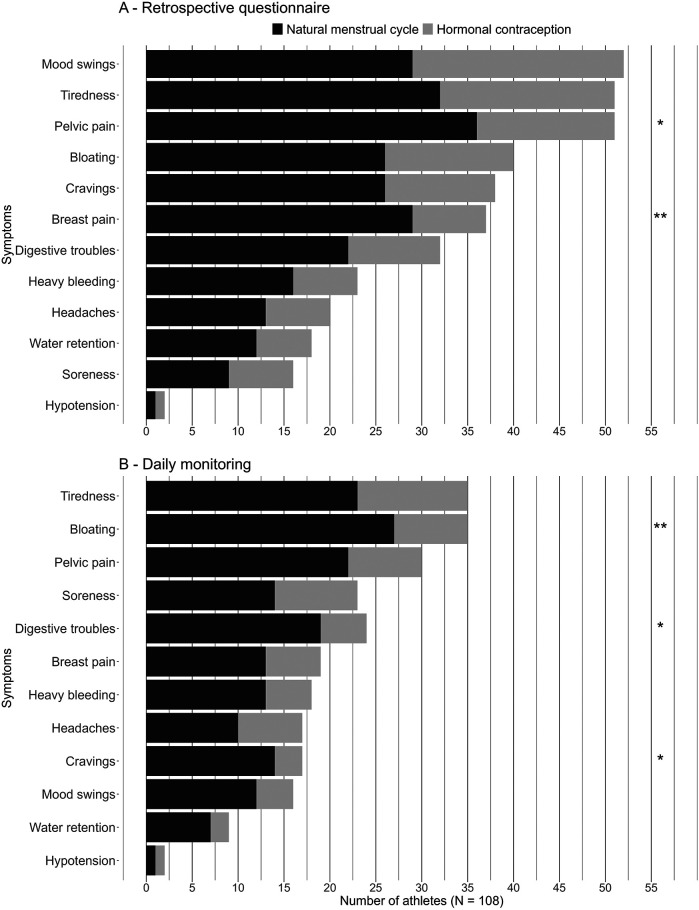
Distributions of reported symptoms by naturally menstruating and hormonal contraception athletes from the retrospective questionnaire **(A)** and from the daily questionnaire **(B)** *p < 0.05, **p < 0.01, ***p < 0.001.

Overall, the most reported symptoms were mood swings (48.1%), tiredness (47.2%), and pelvic pain (47.2%). In MC group, the most reported symptoms were pelvic pains (57.1%), tiredness (50.8%), and breast pain (46.0%). In HC users, mood swings (51.1%), tiredness (42.2%), and pelvic pain (33.3%) were the most common. Pelvic and breast pains were significantly more common in the MC group than in the HC group (*p* = 0.015, and *p* = 0.002 respectively). The reported symptom frequencies and differences between MC athletes and HC users are represented in Figure 1A.

### 3.3 Daily questionnaire

The average number of symptoms reported regularly experienced was 2.3 ± 2.5 for all athletes. MC athletes reported significantly more symptoms (2.8 ± 2.7, *p* = 0.025) than athletes using HC (1.6 ± 2.0).

Overall, the most reported symptoms were tiredness (32.4%), bloating (32.4%), and pelvic pain (27.8%). Among MC athletes, the most reported symptoms were bloating (42.9%), tiredness (36.5%), and pelvic pain (34.9%). Among HC users, the most frequently reported symptoms were tiredness (26.7%), soreness (20.0%), bloating (17.8%) and pelvic pain (17.8%). Bloating, digestive troubles, and cravings were significantly more common in the MC group (*p* = 0.006, *p* = 0.020, and *p* = 0.030 respectively). The reported symptom frequencies and differences between MC athletes and HC users are represented in Figure 1B.

### 3.4 Comparison between retrospective and daily questionnaire

**Figure 2:**
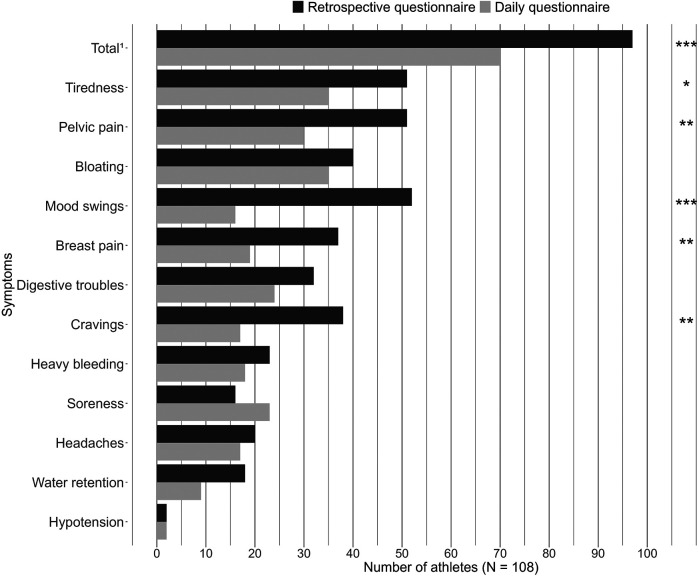
Comparison of reported symptoms depending on methods. ^1^At least one symptom reported in one of the questionnaires. *p < 0.05, **p < 0.01, ***p < 0.001.

As shown in the Figure 2, 89.8% of athletes (*n* = 97) reported regularly experiencing at least one symptom in the retrospective questionnaire, which significantly differed (*p* < 0.001) from the daily questionnaire (64.8%, *n* = 70). Independently of their group, athletes reported significantly more tiredness (*p* = 0.038), pelvic pain (*p* = 0.001), mood swings (*p* < 0.001), breast pain (*p* = 0.009), and cravings (*p* = 0.003) in the retrospective questionnaire than in the daily questionnaire.

### 3.5 Symptoms occurrences during cycles

Over a typical 28-day cycle, at least one symptom was reported on an average of 4.28 ± 5.10 days in regular MC, which was significantly higher than the average of 3.29 ± 4.31 days observed in irregular MC (*p* < 0.001). An average of 2.59 ± 3.43 and 3.29 ± 4.76 symptomatic days were reported in intermittent and continuous HC cycles, respectively. Beyond this overall difference, specific symptoms such as tiredness, soreness, and pelvic pain also showed significant variation across cycle types (Table 1).

**Table 1:** Symptom occurrence (mea*n* ± SD) during a typical 28-day cycle, according to cycle type.

Symptoms	Regular MC	Irregular MC	Intermittent HC	Continuous HC	Global
	(*N* = 272)	(*N* = 66)	(*N* = 183)	(*N* = 33)	*p*-value
Total^a^	4.3 ± 5.1	3.3 ± 4.3	2.6 ± 3.4	3.3 ± 4.8	<0.001^b^
Tiredness	1.4 ± 2.5	1.3 ± 2.9	0.6 ± 1.5	0.7 ± 1.0	<0.001^b^^,^^c^
Bloating	1.3 ± 2.5	0.9 ± 1.8	0.5 ± 1.2	1.3 ± 2.3	0.054
Soreness	0.8 ± 2.0	0.8 ± 2.0	0.7 ± 2.0	1.1 ± 2.6	<0.001^b^
Pelvic pain	0.9 ± 1.5	0.7 ± 1.3	0.4 ± 0.9	1.4 ± 2.7	<0.001^b^
Headaches	0.8 ± 2.3	0.5 ± 1.7	0.6 ± 1.6	0.3 ± 0.7	0.483
Digestive troubles	0.9 ± 2.1	0.4 ± 0.8	0.3 ± 1.2	0.9 ± 1.9	<0.001^b^
Breast pain	0.8 ± 2.0	0.7 ± 1.4	0.2 ± 0.8	1.2 ± 2.9	<0.001^b^
Cravings	0.7 ± 2.1	0.4 ± 0.9	0.2 ± 0.6	0.3 ± 0.6	<0.001^b^
Mood swings	0.5 ± 1.2	0.6 ± 1.4	0.2 ± 0.5	0.3 ± 0.8	<0.001^b^^,^^c^
Water retention	0.5 ± 1.5	0.4 ± 1.1	0.1 ± 0.3	0.2 ± 0.5	0.058
Heavy bleeding	0.3 ± 0.7	0.3 ± 0.7	0.1 ± 0.4	0.3 ± 0.7	<0.001^b^
Hypotension	0.1 ± 0.4	0.0 ± 0.3	0.1 ± 0.5	0.0 ± 0.0	<0.001^b^

a At least one symptom reported during the cycle.

b Significant *post-hoc* comparison: Regular MC vs. Irregular MC.

c Significant *post-hoc* comparison: Irregular MC vs. Intermittent HC.

**Figure 3:**
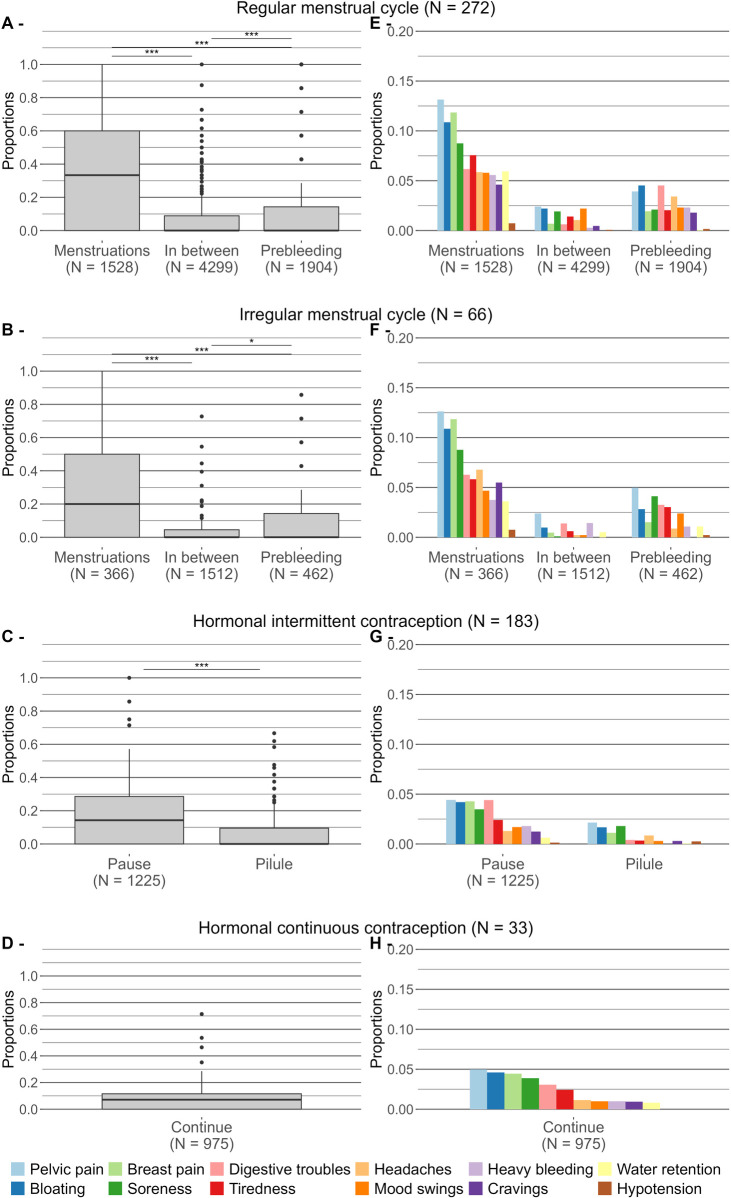
Distribution of athletes according to the proportion of time spent with at least one symptom during cycle phases **(A–D)**, and average proportion of time spent with each symptom during cycle phases **(E–H)** and grouped by cycle type. *p < 0.05, **p < 0.01, ***p < 0.001.

During regular MC, the proportion of symptomatic days was significantly higher during the menstrual phase (34.3 ± 31.4%) compared to during the in-between phase (8.4 ± 16.2%, *p* < 0.001) and prebleeding phase (15.1 ± 27.5%, *p* < 0.001). Additionally, symptoms were significantly more frequent during the prebleeding phase than the in-between phase (*p* < 0.001). During irregular MC, symptoms were significantly more prevalent during menstruations (30.7 ± 30.9%) compared to the in-between phase (5.9 ± 13.9%, *p* < 0.001) and the prebleeding phase (13.0 ± 24.4%, *p* < 0.001). The frequency of symptoms was also significantly higher in the in-between phase than in the prebleeding phase (*p* = 0.045). During intermittent HC cycle, symptoms were significantly more common during the break period (17.9 ± 22.5%) compared to the active hormonal phase (6.5 ± 12.2, *p* < 0.001). In continuous HC cycle, 11.7 ± 17.0% of time was spent with at least one symptom (Figure 3).

### 3.6 Impact of symptoms on well-being

**Figure 4:**
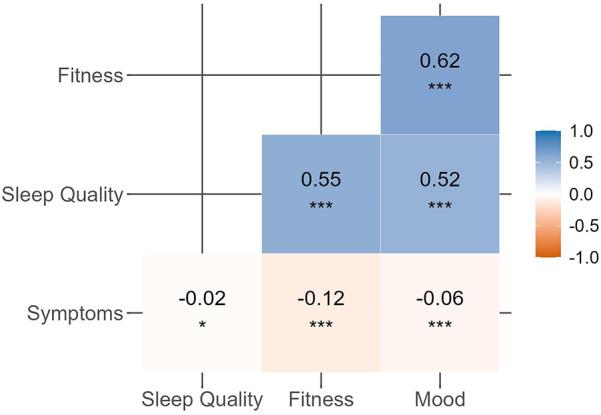
Spearman's correlations between the number of symptoms reported by athletes and well-being indicators (sleep quality, fitness, and mood). *p < 0.05, **p < 0.01, ***p < 0.001.

A significant negative correlation between symptoms and well-being indicators was observed (Figure 4), with Spearman's rho values ranging from −0.02 (sleep quality) to −0.12 (fitness) and −0.06 (mood).

Athletes reported an average fitness score of 6.2 ± 1.8 when they were symptom-free, compared to 5.5 ± 2.2 on days with symptoms (*p* < 0.001). Similarly, the average mood score was 6.7 ± 1.7 without symptoms and 6.4 ± 2.1 on symptomatic days (*p* < 0.001). Average sleep quality was also higher on symptom-free days (6.8 ± 1.8) than on days with symptoms (6.6 ± 2.3, *p* = 0.017).

### 3.7 Impact of symptoms on performance parameters

#### 3.7.1 Football

Training data from 33 football players were analyzed. After removing 265 (13.2%) outliers', data from 1,509 training sessions were available when football players were symptom-free (86.6%) and from 233 training sessions when they reported at least one symptom (13.4%).

There was no significant difference in the total distance covered during trainings: the average was 4,724.3 ± 1,372.9 m when athletes were symptom-free and 4,709.2 ± 1,294.4 m when athletes reported at least one symptom.

**Figure 5:**
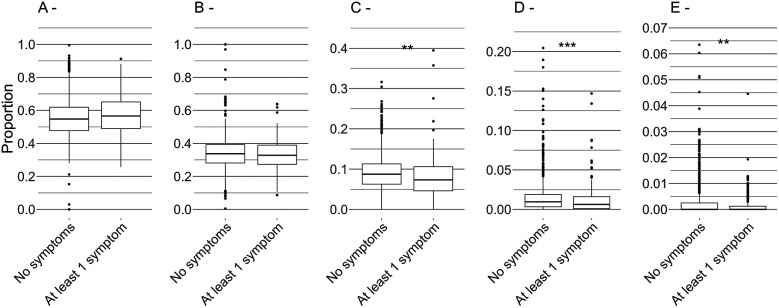
Proportion of total training distance covered at 0-6 km.h^−1^ **(A)**, 6-13 km.h^−1^ **(B)**, 13-19 km.h^−1^ **(C)**, 19–23 km.h^−1^ **(D)**, and above 23 km.h^−1^ **(E)** *p < 0.05, **p < 0.01, ***p < 0.001.

As shown in Figure 5, no difference was observed on distances covered at low speed (0–6 km.h^−1^ and 6–13 km.h^−1^). When football players were symptom-free, they covered significantly more distance at higher speed—between 13 and 19 km.h^−1^ (8.9 ± 4.3% of the total distance, *p* = 0.001), between 19 and 23 km.h^−1^ (1.5 ± 2.0% of the total distance, *p* < 0.001), and above 23 km.h^−1^ (0.2 ± 0.6% of the total distance, *p* = 0.006)—compared to when they reported at least one symptom (8.0 ± 5.2%, 1.2 ± 1.9%, and 0.2 ± 0.4% respectively).

#### 3.7.2 Cycling

Road training sessions from 31 cyclists were analyzed. After removing 92 (5.4%) outliers', data from 1,482 training sessions were available when cyclists were symptom-free (92.0%) and from 129 training sessions when they reported at least one symptom (8.0%).

No significant difference on normalized power output during road training sessions were observed when cyclists were symptom-free (163.4 ± 24.0 W) and when they experienced at least one symptom (164.7 ± 20.2 W).

## 4 Discussion

Based on data from 108 French elite female athletes, covering 554 cycles and totaling 16,491 daily entries, we observed: (i) a significant difference in the prevalence of menstrual symptoms reported using a retrospective vs. a prospective daily questionnaire; (ii) MC athletes reported significantly more symptoms than HC-users whether in a retrospective or daily method; (iii) within the MC group, regular cycles were associated with a higher number of symptomatic days than irregular ones; (iv) a significant impact of symptoms on well-being and high-speed running distance among football players.

In athletic populations, the majority of studies investigating menstrual symptoms in naturally menstruating females or HC users have used retrospective questionnaires or semi-structured interviews (2). Reported prevalence rates of MC/HC-related symptoms vary widely, largely due to methodological differences (2). The findings from our retrospective questionnaire overall aligns with these previous studies, with the most prevalent symptoms being mood swings, tiredness, and pelvic pains.

In our study, the percentage of athletes reporting at least one symptom in a daily survey was notably lower (64.8% vs. 89.8%) than in a retrospective questionnaire. Similarly, the prevalence of specific symptoms such as breast pain, pelvic pain, cravings, mood swings, and tiredness was also lower in daily reporting. To our knowledge, no study has previously compared retrospective and daily data directly.

A retrospective questionnaire is a practical tool for surveying large athlete populations and provides a broad overview of experiences. However, it is prone to memory bias (9) and depends on the individual's self-knowledge and ability to correctly attribute symptoms to the menstrual cycle. Certain symptoms, particularly those with subjective thresholds such as cravings or mood swings, may appear more prominent retrospectively than in real time. Conversely, highly intense or disruptive symptoms, such as pelvic pain or fatigue, may be easier to recall, whereas less bothersome symptoms, such as bloating, may be more consistently captured in daily monitoring.

Another factor that may explain differences is the effort required for reporting. Retrospective questionnaires are filled in once, while daily logs demand sustained engagement. Athletes may be more likely to underreport if asked to log in to report a symptom, as usually is the case with cycle-tracking apps, which rely on users actively deciding to log in only when symptoms occur. In our study, this issue was mitigated by requiring participants to complete the questionnaire every day, regardless of whether symptoms were present. This approach likely increased consistency in daily reports.

Retrospective questionnaires are completed once, whereas longitudinal surveys demand sustained engagement. Athletes may be more likely to underreport symptoms if they are only asked to log them when they occur, as is often the case with cycle-tracking apps. Since this approach relies on athletes actively deciding to report, symptoms may be missed or underreported. In our study, this issue was mitigated by requiring participants to complete the questionnaire every day, regardless of whether symptoms were present. This approach likely improved the consistency of daily reports.

Taken together, these considerations highlight that retrospective and daily questionnaires do not capture the same picture of symptom experience. Retrospective methods may overestimate symptom prevalence due to memory bias, while daily monitoring may underestimate it due to reporting fatigue or omission of mild symptoms. Despite these limitations, daily questionnaires offer the advantage of capturing symptom timing, frequency, and cyclicity more accurately, making them a valuable tool for minimizing recall bias and improving athlete support.

Daily questionnaires also have the advantage of capturing symptoms with greater granularity. Specifically, this type of questionnaire allows for a proper quantification of symptom frequency across different phases of the cycle. Few studies investigate MC/HC-related symptoms (e.g., number, prevalence, frequencies across phases, etc.) through long-term monitoring. McKay et al. (2024) observed similar symptoms prevalence between HC (*n* = 13) and non-HC users (*n* = 11) among female rugby players over a 16-week follow-up (30). They noted more reported symptoms in non-HC users and a positive association between bleeding days and symptoms. McNulty et al. (2023), using a 54-item retrospective survey and a daily questionnaire, found no differences in symptomatology between naturally menstruating recreationally active women and users of combined monophasic oral contraceptive pill (12). However, they reported greater symptom frequency and severity during bleeding using real-time data. Engseth et al. (2024) reported a higher probability of symptoms on bleeding days compared to pre-bleeding and non-bleeding days in winter sports athletes (31). Consistent with these findings, we also observed more frequent symptomatic days during the menstruation, followed by the prebleeding phase. In-between these phases, we observed significantly lower frequency of symptoms. There are various mechanisms for the manifestation of symptoms, including sensitivities to hormonal changes (4). Irregular cycles are characterized by lower hormonal variations (19). However, we observed the same symptomatology pattern in both regular and irregular cycles, although regular cycles were associated with a higher overall number of symptomatic days, highlighting the need for further research to better understand symptom onset. Across intermittent HC cycle, symptoms were significantly more common during the break period than during the active hormonal phase. Unlike McNulty et al.'s study, we found that symptom prevalence differed between natural and HC cycles. These differences are probably due to the more stable hormonal profile in HC use, which leads to fewer symptoms, commonly referred to as negative side effects. The differences between our work and the previously mentioned studies regarding symptom prevalence and patterns in both MC and HC cycles, may be, in addition to the methodology employed, due to differences in follow-up duration and the populations investigated. Interestingly, we found that MC athletes reported a greater number of symptoms than HC users, regardless of whether symptoms were assessed retrospectively or daily. This consistency suggests that both methods are useful for identifying group-level differences. However, retrospective questionnaires do not capture the temporal granularity of symptoms. In contrast, longitudinal monitoring allows for the identification of recurring symptoms and the specific times at which they occur. Such an approach is therefore essential for developing targeted strategies (e.g., nutritional, therapeutic) to mitigate symptoms and support athlete performance. For elite female athletes, this could involve implementing individualized plans, such as adjusting or reducing training loads, according to the type and severity of symptoms. Real-time data analyses allow to consider the unique lifestyle of high-level athletes. For instance, competitive stress, may exacerbate certain symptoms, while weight-category sports can introduce symptoms that are less prevalent outside competition periods, such as those linked to dietary restrictions. Moreover, international travel often brings additional challenges, including jet lag and fatigue, which may further influence symptom occurrence and intensity.

Retrospective surveys have shown that MC-related symptoms can significantly impact well-being. For example, Vannuccini et al. (2020) showed that dysmenorrhea and heavy menstrual bleeding increase stress and decrease mental health scores (32). Heyward et al. (2024) reported a high prevalence of negative wellness effects related to symptoms (6). From our daily questionnaire, we observed a significant negative correlation between symptoms and well-being indicators. Athletes reported lower fitness and mood scores on days when they experienced at least one symptom. These findings are consistent with a previous study by our team, which showed significantly higher well-being scores during the middle of the menstrual cycle—a phase with fewer symptoms—in elite rowers (3).

A recent review highlighted that 24%–100% of athletes reported their performance being negatively affected by their MC, primarily due to MC-related symptoms. However, there is often a disparity between perceived and measured performance (2). A 2020 systematic review and a meta-analysis suggested that exercise performance might be trivially reduced during menstruation compared to other phases (33). Performance outcomes appear more stable across HC cycles but a previous study demonstrated a significant reduction in time spent at high intensity during the withdrawal pill taking phase among combined oral contraceptive users (26). Interestingly these two phases correspond to a high prevalence of symptoms. However, very few studies investigated how the presence of symptoms affects objective performance outcomes. Brown & Duffield (2024) observed that menstrual symptom severity did not impact GPS measured accelerations during football matches in 21 professional players (34). In contrast, our study found a significant reduction in distances covered at >13 km.h^−1^ in female football players experiencing at least one symptom, regardless of their menstrual status (MC or HC use). This discrepancy may be due to difference in sample size, monitoring period or reporting methods. As Brown & Duffield (2024) used a “Menstrual Symptom index”, that considered symptom severity, we focused on the symptom presence or absence. Interestingly, our findings indicate that the impacts are more pronounced on high-speed indices. This aligns with a study by Carling et al. (2024), which reported a lower intensity index during high-effort sessions among elite cyclists in the menstrual phase (27). In cyclists and triathletes, Smith et al. (2024) reported that 92% of athletes experienced at least one perceived MC symptom on at least one race day across two monitored cycles (35). The total number of perceived MC symptoms recorded on race day was positively correlated with race time and negatively correlated with race perception. In our study, we did not observe changes in normalized power among cyclists. The limited number of training sessions with reported symptoms may have constrained our ability to detect differences and to apply the clustering method developed by Carlin et al. (2024), which focuses on high-intensity effort sessions (27). Cyclists may also have implemented adaptive strategies, such as avoiding training on symptomatic days or modifying sessions (e.g., using home trainers), which reduced the available data for analysis. Furthermore, our dataset does not fully account for other training modalities like indoor or track cycling. Finally, differences between individual and team sports should be considered. Team sports like football require sustainable training approaches, whereas cycling allows for more individualized adaptations, making it easier for athletes to adjust their training loads. In contrast to the cited studies that focused on match or race days—comparable to competition settings—our analysis was centered on training sessions. The psychological approach to training and competition differs significantly, as competition often elicits heightened focus and motivation, which may influence the perception and reporting of symptoms as well as their impact on performance. However, analyzing training sessions provides more data and offers a better reflection of an athlete's daily experience. Due to unavailable *in situ* sensors for other sports in our cohort, our analysis primarily focused on cycling and football. For sports where performance cannot be easily quantified, more robust and standardized methods are required (36).

## 5 Limits and strengths

In our daily questionnaire, we did not consider the severity or intensity of symptoms, which represents a limit. This choice was motivated by both technical and practical considerations, as asking elite athletes to rate the severity of each reported symptom would have substantially increased the time required to complete the questionnaire and potentially reduced compliance. Elite female athletes are highly attuned to their bodies, and tend to have a higher pain tolerance than normally active women (37). Consequently, symptoms perceived as mild or not performance-limiting may have gone unreported.

Daily reporting itself also has inherent constraints: it requires sustained engagement, and despite the high compliance rate achieved, some underreporting or reporting fatigue cannot be ruled out. Another limitation is that performance data were only available for sports with objective metrics such as football and cycling, which may limit the generalizability of our findings to athletes from other sports.

Another limitation relates to the determination of menstrual cycle phases. We did not use gold-standard methods such as hormonal verification or ovulation tracking. This choice was intentional, as the primary aim of our study was not to infer hormonal fluctuations, but rather to describe the prevalence and typology of self-reported symptoms according to cycle moments that can be easily monitored in practice. Importantly, assuming a universal hormonal profile for the prebleeding phase would be problematic, as the timing and magnitude of hormonal fluctuations vary both between and within individuals, and many athletes do not present with eumenorrheic cycles. For this reason, our approach prioritized feasibility and ecological validity over precise hormonal phase classification, but this methodological choice should be considered when interpreting the results.

Nevertheless, our study also has important strengths. The high compliance rate of 88.9% achieved in longitudinal monitoring provides confidence in the reliability of the dataset. Moreover, our sample reflects the diversity of real-world athlete profiles, ranging from regular to irregular cycles and from intermittent to continuous HC use, rather than focusing exclusively on the rare eumenorrheic profile. This diversity strengthens the ecological validity of our findings. To our knowledge, this is one of the largest cohorts of elite female athletes monitored over time for menstrual symptoms, offering valuable new insights into their prevalence, variability, and potential impact on performance.

## 6 Conclusion

This study provides new insights into the prevalence, impact, and monitoring of MC/HC-related symptoms in elite female athletes using both retrospective and longitudinal reporting methods. The significant discrepancy observed between retrospective and daily questionnaires suggests that retrospective approaches may lead to overreporting of symptoms, likely due to memory bias or retrospective reinterpretation. This finding emphasizes the added value of closer to real-time, longitudinal data collection in providing a clearer and more accurate picture of symptom frequency and their occurrence across different contraception methods, cycle regularities, or phases. The observed association between increased symptom reporting and reductions in well-being and performance metrics further underscores the need for comprehensive strategies to mitigate these effects. Real-time, longitudinal monitoring can support the development of such strategies by guiding evidence-based and, where appropriate, individualized interventions to help athletes manage symptoms effectively. Future studies should expand on these findings by exploring the underlying mechanisms of symptom reporting differences and by testing the efficacy of targeted interventions across different sports and contexts.

## Data Availability

The raw data supporting the conclusions of this article will be made available by the authors, without undue reservation.
